# Genito-Urinary Surgery

**Published:** 1906-04-21

**Authors:** 


### GENITO-URINARY SURGERY.

*(Continued from page 36.)*

**The Indications and Results of Nephrectomy, especially for Tubercular Disease of the Kidney.**—Rovsing[9] opened a discussion on this subject at the last Congress of the German Surgical Society. Before discussing the methods of ascertaining the presence and functional state of the other kidney, the author considers other questions of importance. These questions are: (1) At what stage in tubercular disease of the kidney is nephrectomy indicated? Some authorities hold that a localised tubercular deposit may heal spontaneously, and therefore constitutional treatment should first be tried. Others maintain that all cases of one-sided disease should be explored, but if the disease is limited to one part of the organ, that part only should be removed. Rovsing, however, strongly supports the view that in all such cases the kidney should be removed completely as soon as the diagnosis is made, for expectant treatment exposes the patients to the risk of dissemination of the disease, and if the kidney is only partially removed, undiscovered disease is very likely to be left behind. (2) How far does extension of the disease to the bladder contraindicate nephrectomy? All agree that limited and slight disease of the bladder often heals well after nephrectomy. But in cases of extensive ulceration spontaneous recovery is not to be looked for, and Rovsing, until recently, found these cases unsatisfactory. Recently he has found even advanced cases yield to repeated washing with 1 in 20 carbolic lotion. His method is to inject 100c.c. of the lotion warmed to 38° C., and allow it to remain in the bladder for five minutes, followed by a second and third similar injection, and this washing is performed every second day. Of eleven cases so treated ten recovered, and the eleventh is well on the way to recovery. Hence the author now holds that advanced bladder disease does not contraindicate nephrectomy. (3) To what extent does associated tubercular disease of the genital organs contraindicate nephrectomy? The author had ten cases with this complication, and nine of these recovered after nephrectomy and orchidectomy or resection of the epididymis, generally done at the same time as the nephrectomy. Hence he does not consider this complication to be a contraindication to nephrectomy. Next Rovsing discusses the methods of estimating the functional capacity of the "second kidney." He condemns as unreliable the results obtained, in conjunction with ureteral catheterisation, by the injection of methylene blue and indigo carmine, or of phloridzin, or the results found by cryoscopy. The author's conclusions, based upon 112 nephrectomies, are as follows:—(1) Renal insufficiency as a cause of death after nephrectomy does not play the important part attributed to it by Casper and Kümmell. (2) The functional capacity of the kidneys cannot be determined by any of the procedures which depend upon a measurement of the amount of excretory work done by the kidneys, because a kidney capable of acting normally may have its functions depressed for the time being; for example, because the other kidney is diseased. (3) Recent improvements in the results of nephrectomy are not due to the introduction of the cryoscopic and phloridzin methods of estimating renal functional capacity. (4) The indications given by cryoscopy of the blood are worthless in judging whether nephrectomy may be performed or not. (5) The phloridzin and urea tests are reliable if positive, but not if negative. (6) When urine from one kidney contains neither albumen, blood nor micro-organisms, the other kidney may be removed. (7) When albumin is present in the urine from one kidney, but pus and micro-organisms are absent, the albuminuria is probably toxic, and is an indication for the removal of the other kidney. In sixty nephrectomies performed since 1901 the author's mortality was only 3.3 per cent.

[8] Amer. Jour. Med. Sci., February, 1906, p. 210. [9] Vide Abst., Edin. Med. Jour., Feb., 1906, p. 171.

